# HIV-Positive Status Disclosure and Associated Factors among HIV-Positive Adult Patients Attending Art Clinics at Public Health Facilities of Butajira Town, Southern Ethiopia

**DOI:** 10.1155/2020/7165423

**Published:** 2020-11-06

**Authors:** Tamirat Melis Berhe, Lire Lemma, Addissu Alemayehu, Desalegn Ajema, Mustefa Glagn, Samuel Dessu

**Affiliations:** ^1^Wachamo University, College of Medicine and Health Science, Department of Public Health, Hosanna, Ethiopia; ^2^Arba Minch University, College of Medicine & Health Sciences, Department of Public Health, Arba Minch, Ethiopia; ^3^Wolkite University, College of Medicine & Health Science, Department of Public Health, Wolkite, Ethiopia

## Abstract

**Background:**

Human immunodeficiency virus-positive status disclosure is the process of informing one's HIV-positive status to others. It is the base for accessing care and treatment programs, attaining psychosocial support, reducing stigma, adhering to treatment, and promoting safer health. Even though different strategies were done in Ethiopia to increase the magnitude of HIV status disclosure among HIV-positive patients, the magnitude is still low. The magnitude of HIV-positive status disclosure was not assessed yet after initiation of the new strategy (test and treat strategy). The aim of this study is to assess the magnitude and factors associated with HIV-positive status disclosure among HIV-positive adults attending antiretroviral therapy clinic at the public health facilities of Butajira town.

**Methods:**

Institution-based cross-sectional study was conducted at public health facilities of Butajira town. A total of 414 study participants were selected by systematic random sampling technique. Data were collected by using pretested interviewer-administered semistructured questionnaire. The collected data were entered into EpiData3.1 and exported to SPSS version 23. Bivariate and multivariable logistic regression analysis was used to identify factors associated with HIV-positive status disclosure. The strength of association was assessed by crude odds ratio and adjusted odds ratio for bivariate and multivariable logistic regression analysis, respectively. Statistically significance was declared at *p* value <0.05 and 95% CI.

**Results:**

The magnitude of HIV-positive status disclosure was 90%. Discussing about safer sex (AOR: 3.5; 95% CI: 1.3, 9.4), viral load suppression (AOR: 4; 95% CI: 1.5, 10.1), having good ART adherence (AOR: 6; 95% CI: 2.4, 14.0), receiving counseling (AOR: 2.5; 95% CI: 1.01, 6.3), and perceiving stigma (AOR: 0.25; 95% CI: 0.09, 0.60) were the independent factors associated with HIV-positive status disclosure.

**Conclusion:**

Although the majority of the participants (90%) of them disclosed their HIV-positive status, lack of disclosure by few people can tackle HIV prevention and control programs. Health programs could improve disclosure of HIV-positive status by providing counseling service, strengthening adherence of antiretroviral therapy, suppressing viral load, and avoiding (reducing) stigma on HIV-positive patients by their community.

## 1. Background

Since the starting of HIV epidemic, above 70 million people have been infected with HIV and 35 million people died worldwide. There had been 36.9 million people having HIV in their blood. Africa (WHO region) is one of the HIV prevalent regions, with 4.1% of the population living with HIV and accounts for 66% of people living with HIV at the end of 2017 [1]. Ethiopia has a large and very vulnerable population that there were more than half million (729, 089) HIV-positive population, 21,606 new HIV infections, and 10,960 deaths in 2018 [2].

HIV-positive status disclosure refers to the act of informing the HIV status of an infected person with or without consent to other people [3]. Disclosure of HIV-positive status plays an important role in strengthening ART adherence and prevention of HIV [4, 5]. It is useful for both the infected individual and the community. It inspires people to seek HIV testing and counseling, change behavior, and decrease the prevalence of HIV [6].

The magnitude of HIV-positive status disclosure in developing countries is lower than in developed countries. The magnitude of HIV-positive status disclosure in developing countries ranged from 16.7% to 86% with the average of 49%. This indicates 50% of people living with HIV/AIDS in developing countries disclosed their HIV-positive status to other person. But, in developed countries, the average magnitude of HIV-positive disclosure status was 79% [7].

The disclosure level of 56.3% was documented by the study conducted at the Mityanaa district hospital of Uganda [8]. According to the study conducted in Southwest Ethiopia; of the total respondents, 37.6% had disclosed their HIV-positive status in general. The failure of people infected with HIV to disclose their HIV-positive status can expose their sexual partner and other relatives that have close contact with them to be infected by the virus [9]. Nondisclosure of HIV-positive status to someone has many consequences like distress, loneliness and social isolation [10], lack of support, infection from their partners with a new type of HIV strain [11], refuse to initiate ART, poor adherence of ART, poor utilization of condom, and chance of mother-to-child transmission of HIV [7]. The aim of this study was to assess the magnitude and factors associated with HIV-positive status disclosure among ART patients attending public health facilities of Butajira town, Southern Ethiopia, 2019.

## 2. Methods

### 2.1. Study Setting and Design

This study was conducted at public health facilities of Butajira town, Gurage Zone, Sothern Ethiopia, 135 km south from Addis Ababa, located at the base of the Zebidar massive in the Gurage Zone of the Southern Nations, Nationalities and Peoples' Region. The town has an estimated total population of 50,290 (24,642 males and 25,648 females). The town has one general hospital, one private hospital, one health center, ten private clinics, and five health posts. ART services provided both at Butajira General Hospital and Butajira Health Center with average monthly attendant of 1021 ART follow-up patients. An institution-based cross-sectional study was conducted from March 01, 2019, to March 30, 2019, on ART clients. The study populations were all HIV-positive adult patients who attending ART clinic for ART treatment at public health facilities of Butajira town.

### 2.2. Sample Size and Sampling Procedure

The sample size was determined by using a single population proportion formula by considering the following assumptions: 95% CI, 5% margin of error, and magnitude of HIV-positive status disclosure in Jimma Specialized Hospital, Ethiopia, (p) which is 37.6% [12]. Accordingly, the calculated sample size with 15% nonresponse rate for this study was 414. The total number of adults who were on ART at the public health facilities of Butajira town was 945 (813 from Butajira General Hospital and 132 from Butajira Health Center). The sample size was allocated proportionally for the hospital and health center and selected by using systematic random sampling. Since total sample size for this study was 414, *k* = 945/414 = 2.28 = 2. Lottery method was used to determine every 1^st^ or the 2^nd^ case. Therefore, every 1^st^ client from the first two patients who came for ART service was selected systematically as the study participants.

### 2.3. Operational Definition

#### 2.3.1. HIV-Positive Status Disclosure

Informing one's HIV-positive status to another person (sexual partner, families or friends, or others) [12].

#### 2.3.2. Current Viral Load Suppression

The last (recent) viral load tested was less than 1000 viral copies per milliliter of blood sample [13].

### 2.4. Data Collection Tools and Procedure

The standardized data collection tool was developed through reviewing of related literature [14–16]. It contains information on sociodemographic factors, individual factors, and disease-related factors. Perceived stigma was assessed by the stigma assessment tool which contains 10 stigma assessment questions.

First, the questionnaire was prepared in English, translated to Amharic and then back to English. A week before the beginning of actual data collection, a pretest was done on 5% of the sample size in Agena town. The data were collected by six case managers/adherence supporters and supervised by two public health officers who were trained on HIV/AIDS comprehensive management and care. Training was given for data collectors and supervisors on how to manage the data collection process. Those data collectors and supervisors who work at Butajira General Hospital were assigned as data collectors of Butajira Health Center and vice versa.

The data were collected by face-to-face interviews and reviewing patient cards by using interviewer-administered semistructured questionnaire.

### 2.5. Data Processing and Analysis

Data were entered into Epidata version 3.1 and then exported to SPSS version 23 for further analysis. The descriptive analysis like percentage, frequency, and mean was calculated. Bivariate and multivariable logistic regression analysis was used to identify associations between dependent and independent variables. In bivariate analysis, variables with *p* ≤ 0.25 were entered into multivariable logistic regression analysis. The Hosmer–Lemeshow test was used to check the appropriateness of the model for analysis. The possible effects of confounders were controlled through multivariable logistic regression analysis. The association between the explanatory and dependent variables were assessed at the *p* value of 0.05. The variables that show *p* value <0.05 were declared as statistically significant variables in multivariable logistic regression analysis. The degree of association between independent and dependent variables was assessed using crude odds ratio (COR) and adjusted odds ratio (AOR) for bivariate and multivariable logistic regression, respectively, with 95% confidence interval.

## 3. Results

### 3.1. Sociodemographic Characteristics

A total of 405 ART patients participated in this study were giving the response rate of 97.8%. The age of respondents range from 19 to 70 years with the mean age of 38 ± 10 years. Of the total participants, 274 (67.7%) were females. Majority 323 (79.8%) of respondents were from urban areas. About 249 (61%) of the study participants were from Gurage ethnic group. More than half (216, 53.3%) of the respondents were Orthodox Christian. One hundred sixty five (37.7%) did not attend formal education. Two hundred ten of the respondents (51.9%) were married (see Table 1).

### 3.2. Disease-Related Characteristics of Study Participants

From a total of 393 respondents who had tested for current viral load, 313 (80%) had current suppressed viral load (<1000 copies/ml) and 213 (59%) had low CD4 count at ART start. Majority (331, 82%) of the respondents had good ART adherence (see Table 2).

### 3.3. Individual Characteristics of Study Participants

From a total of 405 respondents, 303 (75%) had sexual partners. Among those who had sexual partners, 279 (92%) know their sexual partner status (see Table 3)

### 3.4. Magnitude of HIV-Positive Status Disclosure

**Figure 1:**
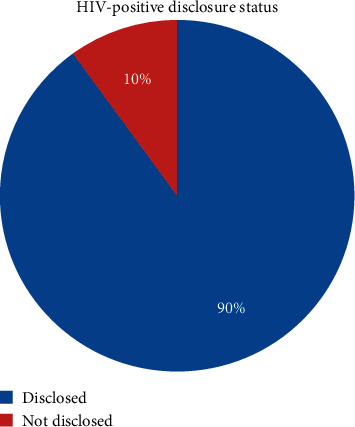
-Magnitude of HIV-positive status disclosure among adult ART patients attending ART clinics at public health facilities of Butajira town, Southern Ethiopia, 2019 (*n* = 405).

Among the study participants, 90% of them disclosed their HIV-positive status (see Figure 1).

### 3.5. Factors Associated With HIV-Positive Status Disclosure

Bivariate analysis was performed to assess factors which were significantly associated with HIV-positive status disclosure. The bivariate analysis showed that discussing about safer sex, viral load suppression, WHO stage at ART start, ART adherence, presence of comorbid condition, receiving counseling, perceived stigma, CD4 count at ART start, current CD4 count, general health condition of the patient, having sexual partner, and belonging to the HIV/AIDS support group were variables which were a candidate for multivariable binary logistic regression analysis.

In multivariable logistic regression analysis, discuss about safer sex, viral load suppression, having good ART adherence, receiving counseling service about HIV-positive status disclosure, and perceived stigma were factors significantly associated with HIV-positive status disclosure. The odds of HIV-positive status disclosure in ART patients who had a free discussion about safer sex were 3.5 times more likely to disclose their HIV-positive status than their counterparts (AOR: 3.5; 95% CI: 1.3, 9.4). ART patients who had good ART adherence were 6 times more likely to disclose their HIV-positive status than those who had poor ART adherence (AOR: 5.9; 95% CI: 2.4, 14). Being in HIV viral load suppression is 4 times more likely to disclose HIV-positive status than being in HIV viral load non suppression (AOR: 3.9; 95% CI: 1.5, 10). Study participants those who perceived stigma were 75% times less likely to disclose their HIV-positive status than their counterparts (AOR: 0.25; 95% CI: 0.10, 0.6) (Table 4).

## 4. Discussion

This study had assessed the magnitude of HIV-positive status disclosure and factors associated with it among people living with HIV/AIDS attending ART clinic in the public health facilities of Butajira town, Gurage Zone, Southern Ethiopia. From the total respondents, 365 (90%) have disclosed their HIV-positive status to another person.

This finding is lower than studies conducted in National Hospital of Abuja, Nigeria (95%), and Northeastern Nigeria (97.5%) [17, 18]. This might be because of the difference in economic status, culture, and ART service delivery programs in Ethiopia and Nigeria. The difference might also be attributed to there were support group service providers at ART centers for HIV-positive patients who were enrolled in HIV\AIDS care and treatment center of Nigeria.

But this finding is higher than studies conducted in Jima Specialized Hospital, Ethiopia (37.6%), Axum Health Facilities, Ethiopia (80.1%), and Ambo Hospital, Ethiopia (86.2%) [12, 16, 19]. The possible explanation for this difference might be because of the collaboration work of governmental and nongovernmental organizations working in public health facilities of Butajira town. There were four nongovernmental projects which work on activities like providing counseling service, partner notification, family index case finding, and adhering the patient for ART service. Four NGOS working in one project might be the justification for this difference. Study time difference might be the other possible explanation. The difference might also be attributed to the initiation of a new program (test and treat strategy).

This study revealed that receiving counseling about HIV/AIDS causes more likely to disclose HIV-positive status than not receiving counseling about HIV-positive status disclosure. It is consistent with the study conducted at Bale Zone Hospitals, Ethiopia; Woldia Hospital, Ethiopia, and Mityana Hospital, Uganda, in which those who received counseling were more likely to disclose their HIV-positive status than those who did not receive counseling service about HIV status disclosure [8, 11, 20]. In another study conducted in Kisarawe Hospital, Tanzania, those who did not receive counseling on HIV/AIDS status disclosure were less likely to disclose their HIV-positive status than those who received HIV-positive status disclosure counseling [21]. The possible explanation might be due to the fact that receiving counseling about HIV/AIDS encourages disclosure, increase awareness, and also bring behavioral change about disclosure. As a result, individuals were able to overcome feelings of shame which facilitated disclosure of HIV-positive status because the patients might knew about the benefits of HIV-positive status disclosure. The other justification might be as patients started ART treatment immediately, they would had frequent contact with health professionals indeed will expose to receive counseling services from health professionals.

This study showed that those who had a discussion about safer sex were more likely to disclose their HIV-positive status than those that did not discuss about safer sex. It is consistent with a study conducted at Jimma town, Ethiopia, in which those who had a discussion about safer sex were more likely to disclose their HIV-positive status than their counterparts [22]. This might be due to the fact that discussion about safer sex may encourage them to have open communication and freedom to disclose their HIV-positive status.

This finding revealed that those who had good ART adherence were more likely to disclose their HIV-positive status than those who had poor ART adherence. This finding is consistent with a study conducted in Tigray, Ethiopia, and Sokodé Regional Hospital, Togo, in which those who had good ART adherence disclosed their HIV-positive status more likely compared to those who had poor ART adherence [14, 23]. This might be due to the fact that adhering to ART might enhance adherence to service provided at health facilities (emphasized on HIV status disclosure), which in turn increases awareness on HIV-positive status disclosure.

This study showed that study participants who had viral load suppression were more likely to disclose their HIV-positive status than those who had a nonsuppressed viral load. It is consistent with the study conducted in the United Kingdom which showed that those who had suppressed viral load were more likely to disclose their HIV-positive status than those who had a nonsuppressed viral load. This might be due to the fact that those who had suppressed viral load had a good adherence and contact with health professionals frequently [24].

This study revealed that ART patients that perceived stigma were less likely to disclose their HIV-positive status than those who perceived stigma. It is consistent with a study conducted in Sydney, Australia, in which those who perceived stigma were less likely to disclose their HIV status than their counterparts [25]. The possible justification might be that the stigmatized patients might fear consequences of disclosure like depression, social withdrawal, psychological stress, and loss of support from families.

## 5. Limitations of the Study

This study was based on self-reporting of the disclosure status which might overestimate the outcome variable because of social desirability bias. The finding may not be inferences to the general population since it was facility-based study. The cross-sectional study design has weaknesses in establishing temporal relationship. The wealth index was not assessed.

## 6. Conclusion

The magnitude of HIV-positive status disclosure in public health facilities of Butajira town is good. Receiving counseling on HIV-positive status disclosure, free discussion about safer sex, viral load suppression, having good ART adherence, and perceiving stigma are independent factors associated with HIV-positive status disclosure.

## Figures and Tables

**Table 1:** Sociodemographic characteristics of ART patients attending public health facilities of Butajira town, Southern Ethiopia, 2019 (*n* = 405).

Variables	Frequency	Percent
*Residence*		
Urban	323	79.8
Rural	82	20.2

*Age in years*		
18–24	26	6.4
25–34	107	26.4
≥35	272	67.2

*Sex*		
Male	131	32.3
Female	274	67.7
*Ethnicity*		
Gurage	249	61
Siltae	53	13
Amhara	56	14
Hadya	40	10
Others	7	2

*Religion*		
Orthodox Christian	216	53.3
Muslim	101	24.9
Protestant Christian	88	21.8

*Educational status*		
No formal education	165	40.7
Primary education	151	37.3
Secondary education	57	14.0
College and above	32	8.0

*Occupation*		
Employed	310	76.5
Unemployed	95	23.5

*Marital status*		
Married	210	51.9
Unmarried	44	10.9
Divorced	65	16.0
Widowed	86	21.2

*Monthly own income* (*ETB*)		
0–500	118	29.1
501–1000	133	32.9
>1000	154	38.0

**Table 2:** Individual and disease-related factors of ART patients attending public health facilities of Butajira town, Southern Ethiopia, 2019 (*n* = 405).

Variables	Categories	Frequency	Percent
CD4 count at ART start (cells/ml)	<250	213	59
	≥250	148	41

Current CD4 count (cells/ml)	<250	31	8
	≥250	330	92

Current viral load (copies/ml)	<1000	313	80
	≥1000	80	20

WHO stage at ART start	I–II	268	66
	III–IV	137	34

Current WHO stage	I–II	380	94
	III–IV	25	6

Duration of ART treatment started (in years)	<l	78	19
	1–2	42	10
	>2	285	71

General health	Worked	391	97
	Ambulatory	14	3

ART adherence	Good	331	82
	Poor	74	18

**Table 3:** Individual and psychosocial factors of adult ART patients attending public health facilities of Butajira town, Southern Ethiopia, 2019 (*n* = 405).

Variables	Categories	Frequency	Percent
Have sexual partner	Yes	303	75
	No	102	25

Know status of sexual partner	Yes	279	92
	No	24	8

Status of sexual partner	Positive	237	85
	Negative	42	15

Belongs to support group	Yes	98	24
	No	307	76

Discuss about safer sex	Yes	247	60
	No	158	40

Receive counselling	Yes	254	63
	No	151	27

Have knowledge on importance of disclosure	Yes	273	58.5
	No	168	41.5

Perceived stigma	Yes	116	29
	No	289	71

Perceived discrimination	Yes	27	7
	No	378	93

History of sexual intercourse with in previous six month	Yes	312	77
	No	93	23

Utilize condom	Yes	253	81
	No	59	19

**Table 4:** Multivariable logistic regression analysis for factors associated with HIV-positive status disclosure among ART patients attending ART clinic in public health facilities of Butajira town, Southern Ethiopia, 2019 (*n* = 405).

Variables	Variables disclose HIV-positive status		COR (95% CI)	AOR (95% CI)
	Yes	No		
*Discuss about safer sex*				
Yes	235	12	4 (2.07, 8.57)^∗∗^	3.5 (1.34, 9.45)^∗∗^
No	130	28	1	1

*Viral load*				
Suppressed	290	23	3.4 (1.71, 6.74)^∗∗^	3.9 (1.52, 10.11)^∗∗^
Not suppressed	63	17	1	1

*ART adherence*				
Good	307	24	3.5 (1.76, 7.05)^∗∗^	5.9 (2.41, 14.54)^∗∗^
Poor	58	16	1	1

*Receive counselling*				
Yes	239	15	3.1 (1.60, 6.212)^∗∗^	2.5 (1.01, 6.30)^*∗*^
No	126	25	1	1

*Perceived stigma*				
Yes	93	23	0.25 (.13,.49)^∗∗^	0.25 (0.10, 0.63)^∗∗^
No	272	17	1	1

^*∗*^  = variables which have *p* value 0 .05–.01; ^∗∗^ = variables which have *p* value < 0.01.

## Data Availability

The data sets used/or analyzed during the current study are available from the corresponding author on reasonable request.

## Abbreviations

AOR: Adjusted odds ratio
HIV: Human immunodeficiency virus
ART: Antiretroviral therapy
AIDS: Acquired immunodeficiency syndrome.
